# sBioSITe enables sensitive identification of the cell surface proteome through direct enrichment of biotinylated peptides

**DOI:** 10.1186/s12014-023-09445-6

**Published:** 2023-12-05

**Authors:** Kishore Garapati, Husheng Ding, M. Cristine Charlesworth, Yohan Kim, Roman Zenka, Mayank Saraswat, Dong-Gi Mun, Sandip Chavan, Ashish Shingade, Fabrice Lucien, Jun Zhong, Richard K. Kandasamy, Akhilesh Pandey

**Affiliations:** 1 Manipal Academy of Higher Education (MAHE), Manipal, Karnataka India; 2 Institute of Bioinformatics, International Technology Park, Bangalore, Karnataka India; 3 Department of Laboratory Medicine and Pathology, Mayo Clinic, 200 First Street SW, Rochester, MN 55905 USA; 4 Proteomics Core, Mayo Clinic, Rochester, MN USA; 5 Department of Urology, Mayo Clinic, Rochester, MN USA; 6 Department of Immunology, Mayo Clinic, Rochester, MN USA; 7 Department of Quantitative Health Sciences, Mayo Clinic, Rochester, MN USA; 8 Center for Individualized Medicine, Mayo Clinic, Rochester, MN USA

**Keywords:** BioSITe, Surfaceome, Biotinylation

## Abstract

**Background:**

Cell surface proteins perform critical functions related to immune response, signal transduction, cell–cell interactions, and cell migration. Expression of specific cell surface proteins can determine cell-type identity, and can be altered in diseases including infections, cancer and genetic disorders. Identification of the cell surface proteome remains a challenge despite several enrichment methods exploiting their biochemical and biophysical properties.

**Methods:**

Here, we report a novel method for enrichment of proteins localized to cell surface. We developed this new approach designated surface Biotinylation Site Identification Technology (sBioSITe) by adapting our previously published method for direct identification of biotinylated peptides. In this strategy, the primary amine groups of lysines on proteins on the surface of live cells are first labeled with biotin, and subsequently, biotinylated peptides are enriched by anti-biotin antibodies and analyzed by liquid chromatography–tandem mass spectrometry (LC–MS/MS).

**Results:**

By direct detection of biotinylated lysines from PC-3, a prostate cancer cell line, using sBioSITe, we identified 5851 peptides biotinylated on the cell surface that were derived from 1409 proteins. Of these proteins, 533 were previously shown or predicted to be localized to the cell surface or secreted extracellularly. Several of the identified cell surface markers have known associations with prostate cancer and metastasis including CD59, 4F2 cell-surface antigen heavy chain (SLC3A2) and adhesion G protein-coupled receptor E5 (CD97). Importantly, we identified several biotinylated peptides derived from plectin and nucleolin, both of which are not annotated in surface proteome databases but have been shown to have aberrant surface localization in certain cancers highlighting the utility of this method.

**Conclusions:**

Detection of biotinylation sites on cell surface proteins using sBioSITe provides a reliable method for identifying cell surface proteins. This strategy complements existing methods for detection of cell surface expressed proteins especially in discovery-based proteomics approaches.

**Supplementary Information:**

The online version contains supplementary material available at 10.1186/s12014-023-09445-6.

## Introduction

The plasma membrane of eukaryotic cells is a dynamic barrier between the contents of the cell and its immediate environment and is home to a large number of proteins [1]. Surface proteins are important determinants of cell identity with roles in cell recognition, adhesion, migration, intercellular communication and signal transduction [2]. While the membrane proteome includes proteins that are localized to the plasma membrane by various mechanisms including single- and multi-pass transmembrane domains, lipid anchors and glycosylphosphatidylinositol (GPI) anchors, the surface proteome is a subset of these proteins defined by the presence of at least one amino acid exposed to the external surface of the cell [3]. The analysis of this subproteome has a wide range of applications in health and disease with a large number of biomarkers and therapeutic targets in neurodegenerative diseases, cancers, and autoimmune conditions being localized to the cell surface [4–6].

Although ~ 30% of genes are projected to encode plasma membrane proteins, these proteins are not detected as often in proteomic profiling studies [7, 8]. This underrepresentation has been attributed to the relatively lower abundance of these proteins as well as to several biochemical and sample preparation considerations. These include the hydrophobic nature and fewer tryptic cleavage sites in membrane transmembrane domains, abundant and complex post-translational modifications (e.g., glycosylation) and the dynamic nature of surface protein abundance and localization across cell states [1, 9, 10]. Several strategies have been used to overcome these difficulties through enrichment of membrane proteins by exploiting their unique properties. These include ultracentrifugation (based on differential density of subcellular fractions) [11], phase-separation (based on physical properties of lipids) [12], lectin- or antibody-mediated enrichment (based on affinity) [13] and chemical labeling followed by affinity purification (based on specific reactivity or extracellular chemical groups) [14]. Addition of a biotin tag to sugar chains of membrane glycoproteins or to extracellular lysine residues followed by pulldown with streptavidin analogs is often employed to study the cell surfaceome [15, 16]. Biotinylation reagents employed in such methods can be cleavable or non-cleavable, depending on the application and method of detection. While these methods successfully enrich membrane (or surface) proteins, they often suffer from contamination with cytoplasmic and organellar proteins to varying degrees. The specificity of several of the available methods is further reduced by the enrichment of intracellular interactors of transmembrane proteins. Additionally, they are often limited by the number of surface proteins that bear the reactive groups being targeted, e.g., the selective enrichment of glycoproteins while precluding the enrichment of non-glycosylated cell surface proteins.

We report the development of surface Biotinylation Site Identification Technology (sBioSITe), a sensitive and reliable method for the enrichment of cell surface proteins by adapting the Biotinylation Site Identification Technology (BioSITe) for immunoprecipitation of biotinylated peptides [17]. We demonstrate that this new method results in confident identification of proteins by localization of extracellular lysine residues and signatures of the biotin tag in fragmentation mass spectra of peptides containing them. This method also enriches secreted proteins that are integral parts of the extracellular matrix or are extracellular binding partners of cell membrane molecules.

## Results

### sBioSITe-based direct identification of biotin-labeled cell surface proteins

**Fig. 1:**
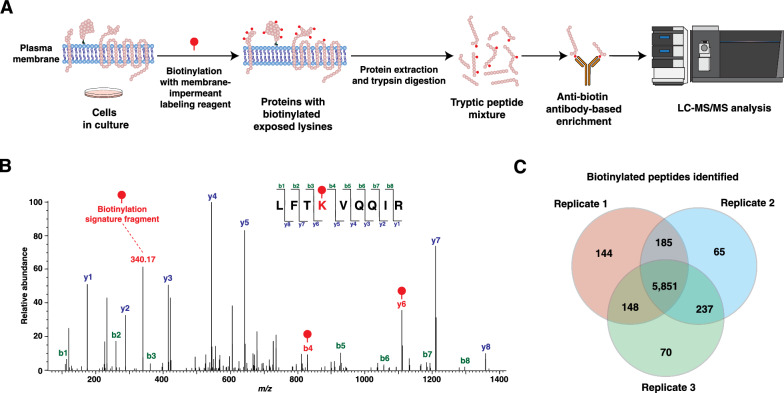
Surface proteome enrichment and direct detection of biotinylated peptides.** A** Experimental strategy. PC-3 cells were treated with a membrane-impermeant non-cleavable biotinylation reagent for preferential labeling of extracellular lysines. Proteins were harvested and digested prior to enrichment of biotinylated peptides using anti-biotin antibodies for LC–MS/MS analysis. **B** Annotated MS/MS spectrum of a representative biotinylated peptide mapped to the GPI-anchored cell surface protein 5′-nucleotidase (NT5E, CD73). The fragment ions in red are diagnostic of the label and localization of the site of biotinylation. **C** The number and overlap of biotinylated peptides identified from the three replicates

Extracellular lysine residues of proteins expressed in PC-3, a prostate cancer cell line, were labeled using a non-cleavable, membrane-impermeant biotinylation reagent in triplicate. Following cell lysis and proteolytic digestion, biotinylated peptides were enriched using our previously published protocol which employs bead-immobilized anti-biotin antibodies [17]. Liquid chromatography–mass spectrometry (LC–MS/MS) analysis was performed to identify cell surface proteins (Fig. 1A). Lysine residues detected with biotin modification were inferred to have been present in the extracellular space during treatment. MS/MS fragment signatures of this modification, including diagnostic *b* and *y* ions as well as a signature fragment of the biotin tag (*m/z* = 340.17), were used to confirm the identification of biotinylation (Fig. 1B). A total of 6700 biotinylated peptides were detected across the three replicates, and a majority of them, i.e., 5851 (87%) were detected and quantified in all replicates (Fig. 1C). These peptides were mapped to 1409 proteins (Additional file 3: Tables S1, S2) and showed consistent levels across the replicates (Additional file 1: Fig. S1).

**Fig. 2:**
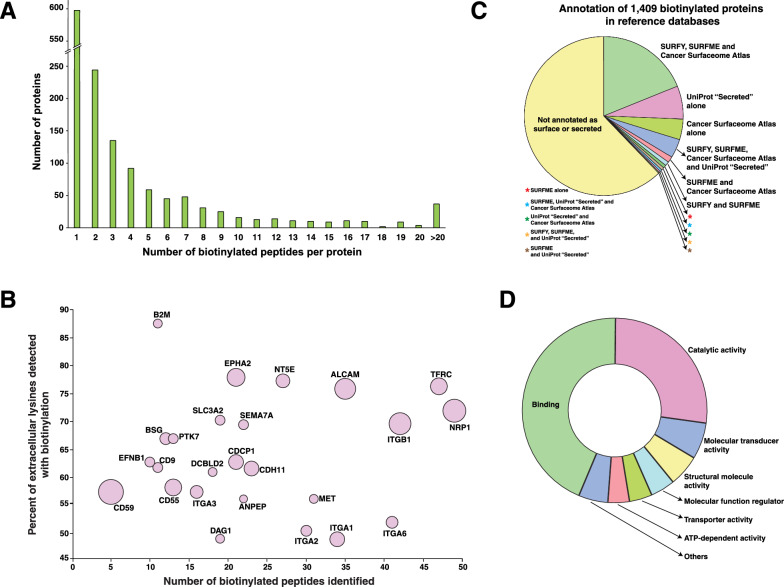
Biotinylation of cell surface proteins.** A** Bar chart showing the number of proteins identified with different numbers of biotinylated peptides. The *y*-axis represents the number of proteins identified by the number of biotinylated peptides shown on the *x*-axis. **B** Bubble plot representing the intensity of biotinylated peptides from the topmost abundant biotinylated proteins. The *x*-axis represents the number of biotinylated peptides identified per protein, and the *y*-axis represents the percent of biotinylation, as the proportion of known extracellular lysines in that protein that were detected with biotinylation. The diameter of each bubble is representative of the intensity of biotinylated peptides from that protein. **C** Pie chart showing the proportions of various proteins that were present in the resources referred to: SURFY (the in silico human surfaceome [3]), the Cancer Surfaceome Atlas [18], SURFME [19] and UniProt for annotations of secreted proteins [20]. **D** Relative proportions of the Gene Ontology annotations for molecular function of the identified biotinylated proteins

A large number of the biotinylated proteins were detected by < 10 biotinylated peptides (Fig. 2A). A total of 587 proteins were detected by a single biotinylated peptide each while 247 proteins were identified by two biotinylated peptides each. To estimate the relative contribution of each protein to the proteome exposed at the cell surface, biotinylation was quantified by the sum of the abundance of all biotinylated peptides derived from that protein. CD59, a GPI-anchored inhibitor of complement-mediated decay, was the single largest contributor of biotinylated peptides by abundance. Although identified by only 5 biotinylated peptides, these were the most abundant peptides from any protein, indicating that this molecule is highly expressed at the surface. The relative contribution of the top 25 proteins to the abundance of biotinylated peptides is shown in Fig. 2B.

We next assessed the robustness of the sBioSITe method for enrichment of proteins that are known to be expressed on the cell surface. We compared the list of biotinylated proteins to three resources: one, SURFY, a machine learning-based in silico surface proteome database [3], two, the Cancer Surfaceome Atlas, a database of genes encoding surface proteins that is based on experimental evidence, computational prediction and database annotation [18] and three, SURFME, a manually curated catalog of surface proteins [19]. Additionally, as many secreted proteins are part of the extracellular matrix which can also be labeled or can act as ligands that bind surface proteins, we made additional annotations for biotinylated proteins with “secreted” as their subcellular location in the UniProt resource [20]. As shown in Fig. 2C, several proteins were accounted for by multiple resources, with 38% of identified proteins annotated as surface or secreted molecules by at least one of these databases. To determine the broad functions of the enriched molecules in cellular processes, we carried out Gene Ontology analysis of biotinylated proteins. As shown in Fig. 2D, the majority of annotated molecules had the molecular functions of “binding” (44%), “catalytic activity” (27%), “molecular transducer activity” (6%) and structural molecule activity (6%) [21].

### Different surface proteins are biotinylated to varying degrees

**Fig. 3:**
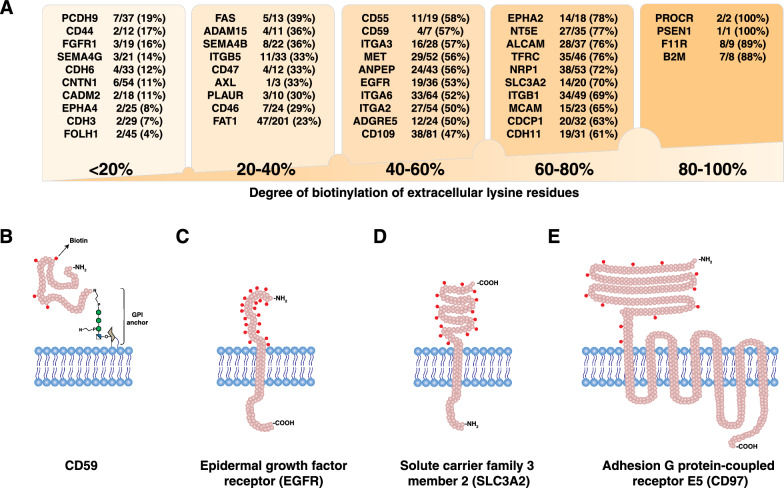
Extracellular lysines are labeled.** A** Representative list of biotinylated proteins grouped by degree of biotinylation, shown along with the ratio of biotinylated lysines to extracellular lysines. **B**–**E** Graphical representation of predicted topology of surface proteins and biotinylated lysine residues, for selected proteins as shown (not drawn to scale)

As the biotinylation reagent employed for sBioSITe preferentially reacts with primary amines, we assessed the number of potential biotinylated sites. For proteins with annotations in the UniProt database of extracellular, transmembrane and intracellular regions, we analyzed the data to determine the proportion of extracellular lysines that were detected with biotinylation [20]. On average, ~ 20% of the extracellular lysine residues were labeled per protein with considerable variation in labeling efficiency. As shown in Fig. 3A, many proteins were identified with high degrees of biotinylation. For example, junctional adhesion molecule A (F11R), involved in the formation of epithelial tight junctions, was identified with eight out of nine (89%) extracellular lysines biotinylated. Presenilin-1 (PSEN1), a multi-pass membrane protein with nine transmembrane domains, was detected with biotinylation at the single lysine residue present in the extracellular region. On the other hand, several molecules involved in cell adhesion, including protocadherin-9 (PCDH9), cadherins 6 and 3 (CDH6, CDH3) and cell adhesion molecule 2 (CADM2) were detected with < 20% of extracellular lysines biotinylated. Interestingly, prostate specific membrane antigen or glutamate carboxypeptidase 2 (PSMA/FOLH1) was identified with only two out of forty-five (4%) extracellular lysines biotinylated. A selection of proteins with different types of attachment to the plasma membrane are shown in Fig. 3B–D and E with representations of biotinylated lysine residues [22].

We wished to determine how the different classically known groups of cell surface proteins were biotinylated. Seventy-eight proteins from the Human Cell Differentiation Molecules, or CD proteins, (HCDM database [23]) were biotinylated. Of the 1409 proteins detected with biotin, twenty-seven were annotated in the UniProt database as being GPI-anchored, and 458 proteins had annotations for transmembrane domains. Of these, 324 had annotations for single transmembrane domains (Additional file 2: Fig. S2A). Four hundred proteins identified were annotated with signal peptide sequences. Nineteen proteins were annotated with the Gene Ontology term of G-protein coupled receptor activity [20, 24], and an additional thirteen proteins were annotated as receptor tyrosine kinases in UniProt or HGNC [25]. Thirty-one proteins were from the solute carrier (SLC) group of proteins. On the other hand, in the subset of 876 biotinylated proteins that had no annotations for surface localization or secretion, 61 proteins had annotations for single transmembrane domains and 44 for multiple transmembrane domains. Thirty-three of these unannotated proteins also had signal sequences (Additional file 2: Fig. S2B). A representative list of the top abundant biotinylated proteins is shown in Table 1 along with the number of extracellular lysines biotinylated in each of them. A complete list of all biotinylated proteins along with the number of biotinylated peptides and surface annotations is given in Additional file 3: Table S2.

**Table 1:** Selected proteins detected with abundant biotinylated peptides and their known association with prostate cancer

	Protein	Number of biotinylated extracellular lysine residues/number of all extracellular lysines (percent)	Association with plasma membrane	Relevance in prostate cancer
1	CD59	4/7 (57%)	GPI-anchored	Associated with disease progression and adverse prognosis [27]
2	Neuropilin-1 (NRP1)	38/53 (72%)	Single-pass type I membrane protein	Promotes progression through EGFR/AKT signaling axis [30]
3	Integrin beta-1 (ITGB1)	34/49 (69%)	Single-pass type I membrane protein	Involved in oncogenic TGF-b signaling [38]
4	CD166 (ALCAM)	28/37 (76%)	Single-pass type I membrane protein	Marker of prostate cancer progression; regulates bone metastasis [28]
5	Ephrin type-A receptor 2 (EPHA2)	14/18 (78%)	Single-pass type I membrane protein	Involved in metastasis [50]
6	Complement decay-accelerating factor (CD55)	11/19 (58%)	GPI-anchored	Promotes cancer cell survival and tumor growth [51]
7	Transferrin receptor protein 1 (TFRC)	35/46 (76%)	Single-pass type II membrane protein	Induces proliferation, migration and invasion in cell lines; ferroptosis-related biomarker [36, 37]
8	CUB domain-containing protein 1 (CDCP1)	20/32 (63%)	Single-pass type I membrane protein	Highly expressed in castration-resistant prostate cancer; therapeutic target [52, 53]
9	Cadherin-11 (CDH11)	19/31 (61%)	Single-pass type I membrane protein	Increases migration and invasion [54]
10	5′-Nucleotidase (NT5E, CD73)	27/35 (77%)	GPI-anchored	Suppresses immune surveillance, prognostic factor [55]
11	Integrin alpha-6 (ITGA6, CD49F)	33/64 (52%)	Single-pass type I membrane protein	Marker of prostate cancer; necessary for self-renewal activity of prostate stem cells [39, 40]
12	Basigin (BSG, CD147)	8/12 (67%)	Single-pass type I membrane protein	Associated with progression; stimulates production of matrix metalloproteases [56, 57]
13	Integrin alpha-2 (ITGA2, CD49b)	27/54 (50%)	Single-pass type I membrane protein	Promotes prostate cancer cell growth within bone [41]
14	Inactive tyrosine-protein kinase 7 (PTK7)	16/24 (67%)	Single-pass type I membrane protein	Prognostic biomarker; predictor of lymph node metastasis [58]
15	4F2 cell-surface antigen heavy chain (SLC3A2)	14/20 (70%)	Single-pass type II membrane protein	Promotes progression via S-phase kinase-associated protein 2 (SKP2) [33]
16	Hepatocyte growth factor receptor (MET)	29/52 (56%)	Single-pass type I membrane protein	Highly expressed in bone metastasis [59, 60]
17	Glypican-4 (GPC4)	25/37 (68%)	GPI-anchored	Interacts with stromal cells; extracellular matrix remodeling, endocrine/paracrine signaling [61]
18	Melanoma cell adhesion molecule (MCAM)	15/23 (65%)	Single-pass type I membrane protein	Involved in metastasis by mediating E-selectin-dependent interaction with bone marrow endothelium [62, 63]
19	Adhesion G protein-coupled receptor E5 (ADGRE5, CD97)	12/24 (50%)	Multi-pass membrane protein	Mediates invasion of prostate cancer cells in association with lysophosphatidic acid receptor 1 (LPAR1) [64]
20	Epidermal growth factor receptor (EGFR)	19/36 (53%)	Single-pass type I membrane protein	Marker of dissemination to bones [29]

## Discussion

Though cell surface proteins are of great importance in basic and translational research, enriching them for analysis is difficult owing to biochemical and analytical reasons. We have developed sBioSITe—a novel method to enrich surface proteins by labeling them with biotin followed by enrichment at the peptide level using anti-biotin antibodies, and detection of peptides with intact biotin tags. A unique feature of the sBioSITe method is the detection of intact biotinylated peptides, indicating extracellular localization of the labeled lysine residue. This gives the identified protein a degree of confidence not afforded by methods that do not allow the detection of intact labeled peptides. Our strategy combined the use of a non-cleavable biotinylation reagent with enrichment using anti-biotin antibodies, enabling us to detect peptides with biotin tags intact. We profiled the surface proteome of PC-3, a prostatic adenocarcinoma cell line and demonstrated sensitive and reliable identification of cell surface proteins. A large number of the identified proteins are known to be localized to the cell surface or secreted into the extracellular space as matrix proteins [26].

### Markers of prostate cancer are abundantly enriched

Many of the biotinylated peptides detected with high abundance were derived from surface proteins that are associated with prostate cancer. A list of selected proteins is shown in Table 1 along with their relevance to prostate cancer. The protein detected with the most abundant biotinylated peptides, CD59, is a GPI-anchored protein which inhibits complement-mediated cell lysis. It has previously been shown to be highly expressed in prostate cancer in association with disease progression and adverse prognosis [27] (Fig. 3B). Another CD marker, CD166 (ALCAM) was detected with 35 biotinylated peptides. CD166 is a marker of prostate cancer progression with a role in metastasis to bone, and is known to be shed from prostatic cancer cells by the action of the sheddase ADAM17, which was also detected with abundant biotinylated peptides [28]. Epidermal growth factor receptor (EGFR), a known marker of dissemination of prostate cancer to bones, was detected with 19 biotinylation sites [29] (Fig. 3C). Neuropilin-1 (NRP1), a single-pass type I membrane protein with a large extracellular domain was detected with 38 biotinylation sites. This protein, a co-receptor for various growth factors, is known to promote prostate cancer progression via modulating EGFR-dependent AKT pathway activation [30–32]. 4F2 cell-surface antigen heavy chain (SLC3A2), a single-pass type II membrane protein which is known to promote prostate cancer progression, was detected with 14 biotinylation sites [33] (Fig. 3D). Ephrin A2 (EPHA2), a tyrosine kinase receptor that is associated with aggressive prostate cancer and adverse prognosis, was detected with 14 biotinylation sites [34]. Adhesion G protein-coupled receptor (ADGRE5, CD97), a seven transmembrane domain-containing protein, was detected with 12 biotinylation sites, all within the large N-terminal extracellular region (Fig. 3E). Similarly, transferrin receptor protein 1 (TFRC), was identified with 42 biotinylation sites. Transferrin receptor levels have been previously shown to be elevated in prostate cancer patients in association with altered iron metabolism, and has been proposed as a ferroptosis-related biomarker [35–37]. Several integrins involved in signaling and metastasis, including integrins beta-1 (ITGB1), alpha-6 (ITGA6, CD49F) and alpha-2 (ITGA2, CD49b) were detected with high abundance [38–41]. It is important to note that the degree of detected biotinylation is influenced by a variety of factors including the topology of proteins and their post-translational modifications (PTMs) [42]. For example, prostate specific membrane antigen or glutamate carboxypeptidase 2 (FOLH1/PSMA) was identified by sBioSITe as a cell surface protein and it is a well characterized diagnostic and potential therapeutic target in prostate cancer [43]. It is a known surface protein with eleven N-glycosylation sites that could heavily influence the number of detected biotinylation sites.

### High sensitivity of surface protein detection

A widely used method for enriching the surface proteome relies on the labeling of exposed sugar residues of glycoproteins [16]. Such a strategy would not identify surface proteins which do not have oligosaccharides available for labeling, including the 436 proteins that are not known to be glycosylated according to UniProt, but were detected by biotinylation in the current study (Additional file 3: Table S1). For example, matrix metalloproteinase-14 (MMP14) is a membrane protein with no known glycosylation sites; it was identified by 16 biotinylated peptides. However, we acknowledge that at least some of the proteins without annotations for N-glycosylation in the UniProt database may actually be glycosylated. Additionally, several proteins have been predicted to be surface localized by machine learning in the in silico surfaceome resource [3]. In our data, we have experimental evidence for some of these predicted proteins to be localized to the cell surface. These include dolichyl-diphosphooligosaccharide-protein glycosyltransferase subunit 1 (RPN1), identified with 10 biotinylation sites, and cation-dependent mannose-6-phosphate receptor (M6PR), identified with 6 biotinylation sites.

Many biotinylated proteins were not annotated as cell surface or secreted molecules in the resources we referred to. Literature curation for some of these proteins revealed that they are known or believed to translocate to the surface or are secreted under some special circumstances. For example, plectin, a cytoskeletal protein involved in tethering the cytoskeleton to membrane complexes, had 114 biotinylated peptides. Although plectin is not annotated as a surface protein, it has been shown to have aberrant localization to the cell surface in some cancers [44]. Several histone proteins including H1.3, H1.2, H1.4 and H1.5 were also identified with biotinylated peptides, consistent with reports that histones are secreted in several cancers including prostate cancer [45, 46]. Nucleolin, a nucleolar protein, was identified by 18 biotinylated peptides; it has been shown to be expressed on the cell surface in association with the actin cytoskeleton in some cancers [47, 48]. Of the 876 proteins not annotated as surface or secreted proteins, 425 were detected with only one biotinylated peptide. As such, many proteins that are otherwise not known to localize to the cell surface but were biotinylated in our data may transiently translocate to the cell surface under specific circumstances. Further experiments in additional cell lines and conditions are needed to validate the surface or extracellular localization of such proteins. Additionally, in a few cases, proteins that are annotated as surface proteins were detected with biotinylation on a few lysines annotated to be “intracellular.” It is pertinent to acknowledge that our databases of the cell surface proteome are perhaps incomplete and accumulating experimental evidence will add more proteins to such lists. We also recognize that the method we report enriches both surface and secreted proteins without distinction and further data analysis may be required to separate the two classes of proteins. In addition, many surface proteins are also known to be secreted (Additional file 3: Table S1); therefore, the labeling of such proteins in the secreted form may lead to biotinylation of lysines that are annotated to be intracellular in their membrane-bound form. Another limitation of this method is that damage to the plasma membrane of even a small proportion of cells during the labeling procedure could potentially lead to the biotinylation of a small number of intracellular proteins, or the biotinylation of intracellular lysines of membrane proteins.

## Conclusions

We have developed sBioSITe as a method for enrichment of biotinylated cell surface proteins with direct detection of labeled peptides. By applying this method for mass spectrometry-based detection of cell surface proteins and their identification by peptides with intact biotin residues, we demonstrate sensitive and confident identification of cell surface proteins. Though the adherent cells used in this study were washed before labeling, the identification of known secreted proteins points to the difficulty of differentially enriching surface proteins from live cells in the context of the extracellular matrix and the surface binding of secreted proteins. We anticipate that this method will be complementary to existing methods of cell surface proteome analysis and is best suited to comparative studies of surface proteomes of cultured cells in different experimental conditions to answer biological questions.

## Methods

### Cell culture and labeling of surface proteins

PC-3 cells were cultured in RPMI 1640 medium with 10% fetal bovine serum (FBS) and 1% penicillin-streptomycin to ~ 80% confluence in 15 cm dishes, with 4 dishes used per replicate. The medium was aspirated and the cells were washed thrice with phosphate-buffered saline (PBS). The cells were then incubated in 10 ml of 0.4 mM of membrane-impermeant amine-reactive biotinylation agent sulfo-NHS-LC-biotin (APExBIO, Boston, MA) in ice-cold PBS for 30 min at 4 °C with gentle swirling of the dish once every 5 min. The solution was then discarded and the reaction quenched twice with 6 ml of 50 mM glycine in ice-cold PBS. The quenching solution was removed and the surface-labeled cells were gently scraped in 500 µl of 50 mM glycine in PBS and collected in a 1.5 ml microcentrifuge tube. The tube was centrifuged at 300×*g* for 5 min and supernatant was discarded. The cells were washed with ice-cold PBS twice and pelleted down by centrifugation at 300×*g* for 5 min and the supernatant removed.

### Protein extraction and digestion

Cell lysis was carried out in 500 µl of modified radio-immunoprecipitation assay (mRIPA) buffer with probe sonication. The lysate was centrifuged at 14,000×*g* for 5 min and the supernatant was collected as the protein fraction, and the pellet discarded. Bicinchoninic acid (BCA) assay was performed to estimate protein amount. 6 mg of protein was taken from each replicate and prepared for digestion. Proteins were reduced with dithiothreitol (Sigma) at a final concentration of 10 mM for 45 min with mild shaking at 37 °C. The sample was then cooled to room temperature and alkylation was carried out with iodoacetamide (Sigma) at a final concentration of 40 mM by incubation at room temperature for 15 min in the dark. Sequencing grade trypsin was added to a final amount of 1:50 (trypsin: total protein), and the samples incubated overnight at 37 °C with mild shaking. The resulting peptides were desalted using C18 reversed phase columns (TopTips, GlyGen), dried in a vacuum centrifuge.

### Enrichment of biotinylated peptides

Biotinylated peptides were enriched using agarose immobilized rabbit anti-Biotin antibody (Bethyl laboratories, A150109A). First, the antibody-coupled beads were washed thrice with PBS and BioSITe capture buffer (50 mM Tris, 150 mM NaCl, 0.5% Triton X-100) thrice. Peptides were dissolved in 600 µl of BioSITe capture buffer. After dissolving peptides, pH was adjusted to neutral (7.0 to 7.5) and colorimetric peptide assay (Pierce) was performed to estimate peptide concentration. Peptides were subsequently incubated with anti-biotin antibody- bound protein G beads for 2 h at 4 °C. The bead slurry was sequentially washed two times with BioSITe capture buffer, two times with 50 mM Tris, and two times with ultrapure water. Biotinylated peptides were eluted four times using elution buffer (80% acetonitrile and 0.2% trifluoroacetic acid in water). The eluent was further cleaned up using C18 reversed phase columns (TopTips, GlyGen) as previously described.

### LC–MS/MS analysis

Previously published LC–MS/MS parameters were used with some modifications. Briefly, samples enriched for biotinylated peptides were analyzed by Orbitrap Exploris 480 mass spectrometer (Thermo Fisher Scientific). Peptides were separated by liquid chromatography on an EASY-Spray column (75 μm × 50 cm, PepMap RSCL C18, Thermo Fisher Scientific) packed with 2 μm C18 particles, maintained at 50 °C. 0.1% formic acid in water (solvent A) and 0.1% formic acid in acetonitrile (solvent B) were used as solvents. Peptides were trapped on a trap column (100 mm × 2 cm, Acclaim PepMap100 Nano-Trap, Thermo Fisher Scientific) at a flow rate of 20 µl/min. LC separation was performed at a flow rate of 300 nl/min and the following gradient was used: equilibration at 5% solvent B from 0 to 10 min, 5–40% sol B from 10.1 to 125 min, 40–95% sol B from 125 to 137 min followed by equilibration for next run at 5% sol B for 13 min. Experiments were done in DDA mode with top 15 precursor ions isolated at a window of 1.2 (*m/z*). Precursors with charge states ranging from + 2 to + 5 were considered for MS/MS events. Normalized collision energy was applied to fragment precursors at energies of 30%. Precursor ions were acquired in the Orbitrap mass analyzer in range of 340–1600 *m/z* at a resolution of 120,000. Fragment ion spectra were detected in Orbitrap mass analyzer with a resolution of 15,000. Automatic gain control (AGC) target value for MS and MS/MS were 10^6^ and 10^5^ and maximum ion injection time were set as 50 ms and 250 ms respectively. Exclude isotopes feature was set to “on” and 20 s dynamic exclusion was applied.

### Mass spectrometry data analysis

Mass spectrometry raw data were searched against UniProt Human Reviewed protein sequences (20,432 entries, downloaded February 1, 2021) by Sequest HT on ProteomeDiscoverer (ver. 2.5., ThermoFisher) [20]. Fully tryptic cleavage specificity with 4 missed cleavages was used and precursor and fragment tolerance were set to 10 ppm and 0.02 Da respectively. Carbamidomethylation of cysteine was set as fixed modification. Oxidation of methionine and protein N-terminal acetylation were set as variable modifications. Biotinylation with NHS-LC-Biotin (+ 339.16 Da) was set as a variable modification at lysine (K). Target false discovery rate (FDR) target was set at 0.01. Minora feature detection was used for precursor ion quantitation calculated using precursor intensity.

### Data analysis

MS/MS spectra of all peptides identified with biotinylated lysine residues were filtered for those containing a signature ion of the biotin tag (m/z = 340.1698 ± 10 ppm) and 22 peptides without this signature were removed from analysis. Peptides identified in all replicates were mapped to proteins and considered for further analysis as described. Gene Ontology analysis of enriched proteins was carried out using PANTHER [21].

### Associated data

The mass spectrometry proteomics data have been deposited to the ProteomeXchange Consortium via the PRIDE [49] partner repository with the dataset identifier PXD044519.

### Supplementary Information

**Additional file 1: Figure S1.**Scatter plots showing consistency of detection of biotinylated peptides. Intensity values of biotinylated peptides from each replicate were log-transformed and plotted against each other in pairs.

**Additional file 2: Figure S2.**Features of identified proteins. A. Number of proteins among all biotinylated proteins grouped by distinguishing features B. Number of biotinylated proteins that have not been annotated as cell surface or secreted proteins with features as shown.

**Additional file 3: Table S1.** Biotinylated peptides detected in common in all replicates. **Table S2.** Biotinylated proteins detected in common in all replicates.
